# Nocturnal exposure to a preferred ambient scent does not affect dream emotionality or post-sleep core affect valence in young adults

**DOI:** 10.1038/s41598-024-60226-z

**Published:** 2024-05-06

**Authors:** Lenka Martinec Nováková, Eva Miletínová, Monika Kliková, Jitka Bušková

**Affiliations:** 1 Department of Chemical Education and Humanities, University of Chemistry and Technology, Prague, Technická 5, 16628 Prague 6 - Dejvice, Czech Republic; 2 National Institute of Mental Health, Topolová 748, 25067 Klecany, Czech Republic; 3 3rd Faculty of Medicine, Charles University, Ruská 87, 10000 Prague 10, Czech Republic

**Keywords:** Dreaming, Hedonic, Olfactory, REM, Smell, Human behaviour, Psychology

## Abstract

Emotions experienced within sleep mentation (dreaming) affect mental functioning in waking life. There have been attempts at enhancing dream emotions using olfactory stimulation. Odors readily acquire affective value, but to profoundly influence emotional processing, they should bear personal significance for the perceiver rather than be generally pleasant. The main objective of the present sleep laboratory study was to examine whether prolonged nocturnal exposure to self-selected, preferred ambient room odor while asleep influences emotional aspects of sleep mentation and valence of post-sleep core affect. We asked twenty healthy participants (12 males, mean age 25 ± 4 years) to pick a commercially available scented room diffuser cartridge that most readily evoked positively valenced mental associations. In weekly intervals, the participants attended three sessions. After the adaptation visit, they were administered the odor exposure and odorless control condition in a balanced order. Participants were awakened five minutes into the first rapid eye movement (REM) stage that took place after 2:30 a.m. and, if they had been dreaming, they were asked to rate their mental sleep experience for pleasantness, emotional charge, and magnitude of positive and negative emotions and also to evaluate their post-sleep core affect valence. With rs < 0.20, no practically or statistically significant differences existed between exposure and control in any outcome measures. We conclude that in young, healthy participants, the practical value of olfactory stimulation with self-selected preferred scents for enhancement of dream emotions and post-sleep core affect valence is very limited.

## Introduction

Mental activity in sleep, also referred to as “sleep mentation”, “mental sleep experience”, or simply “dreaming”^1^, can profoundly affect daytime mood^2,3^, mainly when the experience is negatively emotionally valenced^4^. Emotions appear to be present in a high proportion of recalled dreams^5^ and, for the most part, tend to be negative rather than positive, even in healthy individuals^5,6^. Particularly distressing emotions, such as anxiety, fear, terror, anger, sadness, or frustration^7^, are experienced in bad dreams (which are negatively toned dreams that do not wake the sleeping person up) and in nightmares (which do lead to awakening)^8^. They may distort sleep architecture^9^ and hence impair sleep quality^10^, but also many other aspects of both sleeping and waking life^11^, including well-being^12^. The link between mental sleep experiences and functioning in waking life may nevertheless generalize to all dreams^13–15^.

Given the multifaceted influence of mental sleep experiences on waking life^16,17^, there have been attempts to modulate emotions experienced while dreaming, e.g., through sensory stimulation^18–21^. The sensory modality that readily lends itself to this purpose is olfaction because it is tightly interwoven with affective processing^22,23^. Hedonic value plays a central role in olfactory processing^24^, and odors have been repeatedly shown to influence affective processing even without conscious perception^25,26^. This close association between the sense of smell and emotion has been attributed to their shared neural substrates^27^, which include the amygdala and hippocampus^28–30^ and the prefrontal cortex^31,32^. Hence, one notable aspect of olfactory processing is the ease with which odors acquire their affective value. This usually happens through evaluative conditioning^33,34^, whereby odor stimuli readily assume the tone of the context within which they were experienced^35–38^. This acquired affective value then translates to their effects on affective and cognitive processing on following exposures^39^, which can be inferred from neural, physiological, and behavioral responses^37,38,40–45^.

The capacity of odors to influence mental processing has been utilized in studies of the effects of odor exposure during sleep on sleep mentation. It transpired that odors presented during sleep may affect dream emotions and possibly even dream content^3,4,46,47^. For instance, in 15 healthy young females in rapid eye movement (REM) sleep, Schredl and colleagues^47^ found that the emotional tone of sleep mentation (i.e., “dreams” in the broadest sense^1^) was somewhat more positive upon exposure to generally pleasant phenylethylalcohol (PEA, which has a rose-like odor) compared to generally unpleasant hydrogen sulfide (H_2_S, which smells of rotten eggs). On the other hand, in depressed female inpatients, Vitinius and colleagues^48^ observed no significant effect of exposure to PEA on sleep mentation or mood. Another team that used PEA for REM exposure recruited healthy participants who either liked (N = 7, 4 men) or disliked (N = 8, 2 men) its odor^46^. Their findings were, however, rather surprising in that liking the smell of PEA was linked to a greater degree of negative emotions experienced while asleep under the PEA condition compared to the odorless one. Finally, Sabiniewicz et al.^49^ randomly assigned their participants to either odor (orange, perfume, or laurinal; delivered via a nose clip) or placebo group and investigated whether condition affected sleep quality over a fortnight of odor exposure/non-exposure and another fortnight post-exposure/non-exposure, controlling for odor pleasantness. They found that reports of sleep and dreaming did not differ with respect to group. Participants who found the odors more pleasant felt more rested on night 28 compared to night 1, while those who rated them as less pleasant tended to feel more rested on night 13 compared to night 28^49^.

Odor pleasantness tends to exhibit associations with other perceptual properties, such as familiarity [e.g.,^50^]. Hence, in line with their previous finding, in a later study, Okabe et al.^51^ showed that adolescents who were grouped according to the rated familiarity of PEA (low/high) reported more negative emotions in their dreams in the PEA condition when its odor was perceived as highly familiar. On the other hand, the latter group experienced more positive dreams than the former when no odor was presented to them^51^. Another characteristic linked to olfactory hedonic appraisal is odor intensity^50^. Recently, Okabe and Abe^52^ observed no differences in dream emotionality and emotional tone between participants who perceived the odor of PEA as intense vs. weak.

Currently, the evidence on the influence of smells presented during sleep on sleep mentation is inconclusive, and the links of perceived odor pleasantness or other related perceptual properties with dream characteristics seem mixed. A potential reason might be that researchers have focused on perceptual pleasantness, disregarding odors’ affective value, which tends to be highly idiosyncratic across people and might lie at the core of olfactory effects on dreaming. Despite this, olfactory exposure studies in which individual odor preference was considered are the exception rather than the rule^46,53^. In the present investigation of the capacity of ambient odor to modulate affect, we aimed to make full use of the profound individuality of olfactory preferences. This was accomplished by asking participants to choose from a large set of designer room fragrances one scent that most readily evoked positively-valenced mental associations (e.g., reminded them of a beloved significant other, moment of happiness, treasured object, etc.) or conjured up pleasant imagery. Building on the literature on olfactory associative learning and odor primes, the main objectives of the present study were to test whether (1) dream pleasantness, (2) dream emotional charge and magnitude of (3) positive and (4) negative emotions present in dreams differ upon extended, several hour-long exposure to a subjectively salient pleasant ambient odor compared to a time spent in the same room without odorization. Furthermore, assuming continuity between dreaming and waking life [e.g.,^14,15^], we also tested whether (5) the valence dimension of a person’s post-sleep affect (e.g., feeling happy, optimistic, and relaxed rather than unhappy, pessimistic, and tense) differs between the two conditions. We hypothesized that dreams would be experienced as more emotionally charged, contain a greater degree of positive and a lesser degree of negative emotions, and that the individual’s post-sleep core affect would be more positively valenced upon exposure compared to the odorless condition. Although dreaming is by no means limited to REM sleep^54^, REM mental processing may qualitatively differ from non-REM^55^, particularly in the greater involvement of REM sleep in  the processing of emotional material^56^. Therefore, reports were collected upon awakening from REM.

## Results

### Descriptives

Descriptive statistics of the participant sample are shown in Table 1.

**Table 1:** Absolute frequencies and percentages for education, time of going to bed, dream recall and emotional intensity of dreams, and mean ± SD for age, Beck Depression Inventory-II (BDI-II), State-Trait Anxiety Inventory (STAI X-II), University of Pennsylvania Smell Identification Test (UPSIT) scores, monthly consumption of alcohol and stimulants, and sleep duration on weekdays and the weekend.

	Men (N = 12)	Women (N = 8)	Total (N = 20)
Age	25.8 ± 4.5 (20–35)	24.0 ± 1.7 (22–27)	25.1 ± 3.7 (20–35)
Education			
Elementary	0	0	0
Secondary	6 (50%)	5 (63%)	11 (55%)
Bachelor’s Degree	3 (25%)	2 (25%)	5 (25%)
Master’s Degree	2 (17%)	1 (13%)	3 (15%)
Doctoral Degree	1 (8%)	0	1 (5%)
BDI-II score	3.2 ± 3.2 (0–11)	2.9 ± 2.2 (0–6)	3.1 ± 2.8 (0–11)
STAI X-II score	37.9 ± 8.2 (24–53)	32.4 ± 5.7 (23–39)	35.6 ± 7.6 (23–53)
UPSIT score	31.6 ± 3.6 (25–37)	34.6 ± 1.8 (32–38)	32.8 ± 3.32 (25–38)
Alcohol			
Soft	6.3 ± 6.6 (0–18)	9 ± 10.6 (1–32)	7.4 ± 8.2 (0–32)
Hard	0.9 ± 1.3 (0–4)	0.3 ± 0.5 (0–1)	0.7 ± 1.1 (0–4)
Stimulants			
Coffee	6.5 ± 9.6 (0–28)	14 ± 23 (0–60)	9.5 ± 16.2 (0–60)
Tea	17.9 ± 17.9 (0–60)	18.4 ± 16.9 (0–50)	18.1 ± 17.1 (0–60)
Energy drinks	0.2 ± 0.6 (0–2)	0.4 ± 0.7 (0–2)	0.3 ± 0.6 (0–2)
Cigarettes	4 ± 8.9 (0–30)	0	2.4 ± 7.1 (0–30)
Time of going to bed on weekdays			
Before midnight	10 (83%)	6 (75%)	16 (80%)
At midnight or later	2 (17%)	2 (25%)	4 (20%)
Sleep duration (hours)			
Weekdays	7.7 ± 0.5 (7–8.5)	7.6 ± 0.7 (6.5–8.5)	7.7 ± 0.6 (6.5–8.5)
Weekends	9.0 ± 0.7 (8–10)	8.9 ± 0.6 (8–10)	8.9 ± 0.7 (8–10)
Self-assessed dream recall			
Once a week	6 (50%)	4 (50%)	10 (50%)
More than once a week	6 (50%)	4 (50%)	10 (50%)
Emotional intensity of dreams			
Somewhat intense	8 (67%)	3 (38%)	11 (55%)
Quite or very intense	4 (33%)	5 (62%)	9 (45%)

Alcohol units for soft alcohol represent a small beer (0.33 l) or a glass of wine (0.2 l); for hard alcohol a 0.2 l glass. Units for coffee/tea and energy drinks are cups and bottles, respectively.

### Main results: differences between the exposure and control condition

Wilcoxon signed-rank tests did not reveal any statistically significant differences between the exposure and control condition in dream pleasantness, emotional charge, the magnitude of positive or negative emotions, or the valence dimension of core affect. With rs < 0.20, the practical significance of most of the effects fell well below the recommended minimum effect size that represents a “practically” significant effect^57^, although the comparisons of dream emotional charge and mean positive and negative emotions, respectively, closely missed this mark. For complete results, please see Table 2.

**Table 2:** Absolute (N) and relative frequencies (percentages) for dream recall and odor presence appraisal, mean ± SD, median, and range for dream pleasantness and emotional charge, mean positive and negative emotions, and the valence dimension of post-sleep core affect.

	Odorless control			Odor exposure			Wilcoxon signed-rank test			
	Mean ± SD	Median	Range	Mean ± SD	Median	Range	W	p	r	N
Dream recall (Yes/No)	15 (75%)			15 (75%)						
Reported odor presence (Yes/No)	3 (15%)			12 (60%)						
Dream pleasantness (1–7)	4.47 ± 0.92	4.00	3.00–6.00	4.47 ± 1.13	4.00	3.00–7.00	20.50	0.722	0.07	12
Dream emotional charge (1–7)	3.40 ± 1.96	3.00	1.00–7.00	4.13 ± 1.30	4.00	2.00–6.00	19.00	0.391	0.17	12
Mean positive dream emotions (1–7)	3.22 ± 1.36	3.00	1.50–6.25	3.58 ± 1.71	3.75	1.50–6.00	49.00	0.432	0.16	12
Median positive dream emotions (1–7)	3.27 ± 1.70	3.00	1.00–7.00	3.63 ± 1.89	4.00	1.00–6.00	39.50	0.562	0.12	12
Mean negative dream emotions (1–7)	1.82 ± 1.07	1.50	1.00–4.75	2.15 ± 1.18	1.75	1.00–5.00	43.00	0.370	0.18	12
Median negative dream emotions (1–7)	1.60 ± 1.17	1.00	1.00–5.00	1.90 ± 1.17	1.50	1.00–5.00	27.50	0.546	0.12	12
Core affect valence index (3–27)	16.75 ± 3.34	16.00	12.00–24.00	16.59 ± 3.59	16.00	11.00–23.00	35.00	0.858	0.03	14

### Exploratory comparisons

#### Differences between the randomization groups

In the control condition, there were no practically and/or statistically significant differences between participants depending on their randomization status (i.e., whether the control session was scheduled for their second or third visit). However, it did matter when exposure occurred in terms of the reported magnitude of negative dream emotions and post-sleep core affect valence. Specifically, participants exposed on their third visit reported lower intensities of negative dream emotions (mean ratings: 2.82 vs. 1.56, respectively; Mann–Whitney U = 10.50, *p* = 0.038, r = -0.53, N = 7 vs. 8), as well as higher Valence scores (14.78 vs. 18.63; U = 15.00, *p* = 0.042, r = 0.49, N = 9 vs. 8) than volunteers stimulated on their second visit. In other words, participants whose room was odorized on their third visit experienced less intense negative emotions in their dreams, and felt pleased, glad, and happy to a greater extent than displeased, sad, and depressed, respectively (which were the three adjective pairs loading onto the Valence dimension^58^).

#### Gender differences

Several differences emerged between the female and male participants. Women reported greater emotional charge of their dreams (control: 4.83 ± 1.94 vs. 2.44 ± 1.33; U = 8.00, *p* = 0.022, r = -0.59; exposure: 4.86 ± 1.07 vs. 3.50 ± 1.20; U = 12.00, *p* = 0.054, r = -0.50). Women also reported higher post-sleep core affect valence under exposure (18.38 ± 3.38 vs. 15.00 ± 3.12; U = 15.50, *p* = 0.047, r = -0.48). On their third (but not second) visit, women (compared to men) reported a greater emotional charge of their dreams (5.50 ± 1.23 vs. 2.88 ± 1.13; U = 3.00, *p* = 0.006, r = -0.74). Additional descriptive statistics and exploratory analyses are presented in the Supplementary Material.

## Discussion

In the present study, we aimed to investigate whether spending the night in a sleep laboratory bedroom odorized with a self-selected pleasant odor affects dream pleasantness and emotional charge, the magnitude of positive and negative dream emotions, and the valence of the post-sleep affective state. The underlying assumption was that the olfactory modulation of affect would be afforded by the previous association of the self-selected odor with something in the participants’ experience that they explicitly designated as pleasant^23,59,60^.

We did not find any significant differences in the outcome measures between the odorless control and odor exposure condition. In terms of practical significance, the effect sizes were negligible. However, some emotion-related measures were close to r = 0.20, which some authors the lower limit of what could be called a “small” effect^57^. Hence, we tentatively suggest that the practical utility of exposing oneself to self-selected pleasant odor stimuli while asleep to enhance dream emotionality and post-sleep core affect valence is very limited in young, healthy adults. This is, however, not to say that sensory-based interventions in general and olfactory exposure in particular are of no practical value. These applications have been investigated predominantly within settings where optimization of arousal is desirable, such as in-patient and community health services^61–64^, high-stress academic environments^65^, or even classrooms at lower educational stages^66^. In particular, there is a growing body of research on the use of sensory design in general and sensory rooms in particular in the mental health and social work settings, which has been reported, e.g., to reduce stress and facilitate self-regulation, instill relaxation and augment positive emotions^67–70^. Modified sensory environments are employed to achieve a range of outcomes with diverse clinical populations. The use of soothing ambient stimuli to support adaptive emotion regulation has broad applications. Everyone can benefit from self-care practices that enhance sensory comfort to promote well-being^66,71^. However, we should remember that such olfactory interventions must be highly personalized and that actual exposure likely serves as a mere facilitator of the effects the given individual attributes to the odor in question.

Relatedly, a crucial methodological aspect that is often overlooked is the considerable individual variation in hedonic responses to odors^72–75^. There has been a notable lack of appreciation for the idiosyncrasies of olfactory perception in general and odor hedonics in particular^50^. On the one hand, pleasantness is linked to physicochemical structure of molecules^24,76,77^, with pleasant ones generally being larger and containing oxygen^78^. Arshamian et al.^74^ found substantial global consistency in odor liking across a number of cultures, which was predicted by the physicochemical properties. Some odors may be universally liked, such as sweet and floral ones^24^, although the liking will vary with intensity. The relationship between pleasantness and intensity may be linear for some odors but usually follows an inverted U-curve trend^79–81^. Yet, for an odor to profoundly influence affective processing and behavior, it needs to be perceived as subjectively—rather than “universally”—pleasant^82^, but also to have subjective affective value^83^. This is particularly evident in studies on odor-evoked autobiographical memory and nostalgia (for review, see^84^).

Olfactory exposure studies in which individual odor preference was considered are the exception rather than the rule^46,53^. One of the few exceptions was the study by Villemure, Slotnick, and Bushnell^53^, who reported that mood improved, anxiety decreased, and perceived pain unpleasantness diminished following exposure to a subjectively pleasant odor. In contrast, exposure to one that they designated as unpleasant had the opposite effect.

In the present investigation of the capacity of ambient odor to modulate affect, we aimed to make full use of the profound individuality of olfactory preferences. This was addressed by asking participants to choose from a large set of designer room fragrances one scent that most readily evoked positively-valenced mental associations (e.g., reminded them of a beloved significant other, moment of happiness, treasured object, etc.) or conjured up pleasant imagery. However, the stimuli were selected under a forced-choice paradigm and may not reflect one’s true olfactory preferences, which is one of the current study's limitations. We utilized room scents that were readily available from the odor diffuser manufacturer to keep the perceived intensity at comparable levels across participants and to have complete knowledge of the scents’ chemical composition and odor profile. Also, we only asked the participants to pick the exposure stimuli on their first, adaptation session that was not subject to formal analysis, which was to reduce the effect of the participants’ potential expectancies on the reports collected within the next two sessions that were a week and a fortnight apart from this baseline, respectively. Yet, that may have created a sense of pressure in the participants or feeling overwhelmed at having to deal with a 40-item stimulus set, even though they had been explicitly instructed to take their time as much as they needed and appraise the stimuli at their own pace during the ~ 4-h gap between the check-in and the start of the nocturnal session.

Another issue might have been the prolonged period of continuous olfactory stimulation, which was adopted to mimic the conditions of at-home odor exposure as closely as possible. It may have resulted in olfactory adaptation, which, on the behavioral level, manifests itself as habituation, i.e., reduced responsiveness to continuous or repetitive stimulation^85,86^. Yet, it is unclear whether olfactory adaptation and habituation pose a significant obstacle to olfactory modulation of sleep mentation. In studies that employed advanced olfactometry to minimize these potentially adverse effects, the rates of incorporating olfactory stimuli in sleep mentation were relatively low^46,47,87^.

On a related note, it is not entirely clear whether conscious awareness of odor presence during waking is a shortcoming in studies like the present one or whether it is actually desirable. On the one hand, unmasked odors may be used as cues that may influence participants’ retrospective reports about the preceding sleep period in line with beliefs about the odors and their actions. As but one example, the purported effect of essential oils may be modulated by one’s expectancy or belief about their efficacy^88,89^. On the other hand, reporting odor presence may indicate that the prolonged odor exposure did not result in olfactory adaptation/habituation. There is, nevertheless, no conclusive evidence as to whether these processes interfere with odor-induced modulation of dream emotionality ratings and post-sleep core affect. Conscious odor perception may not be necessary for (some) odors to influence our feelings and actions^25,26^. In our study, we made no effort to mask the odor presence during waking to achieve a more ecologically valid setting that resembled circumstances under which room odorization would occur at people’s homes. As Table 2 suggests, 12 (60%) participants reported that they perceived an odor in the exposure condition, presumably reflecting their conscious awareness of the self-selected scent. Given the way the question was phrased (“Have you noticed any odor in this room?”), an affirmative response might also indicate that any faint background odor in the room was detected. In other words, an affirmative reply was not necessarily a “hit” in the sense that the participant noticed the self-selected scent. Theoretical debates notwithstanding, there were no statistically significant differences in the outcome measures between “hits” and “misses”. This might, however, be attributed to the small N. With effect sizes rs ranging between 0.05 (for dream emotional charge) and 0.48 (for mean positive dream emotions), appraisal of odor presence/absence, which could not be meaningfully included in the present models due to the limited sample size, warrants consideration in future studies.

Related to this, while we had carried out an a priori sample size computation (as detailed in the Participants section), we unfortunately found ourselves unable to meet the target N. This was because, firstly, we had obtained a lower dream recall rate of 75% compared to our previous study^90^, which had a rate of 83%. Secondly, we encountered technical issues during the v-PSG recordings, rendering fewer reports available for analysis. The tight lab schedule prevented us from making up for the lost data within the limited time slot dedicated to the present study. Hence, future studies would benefit from accounting for factors that are difficult to predict and are beyond the experimenters’ control, such as the dream recall rate, and increasing the N accordingly.

## Materials and methods

### Participants

Twenty healthy young adults aged 25.1 ± 3.7 years participated in the study, of which 12 self-identified as male (25.8 ± 4.5 years) and eight as female (24.0 ± 1.7 years). Men and women did not differ in age. The inclusion criteria for participation were (1) being over 18 but less than 35 years of age (which was to reduce the potential confounding effect of olfactory decline that begins towards the end of early adulthood^91^) and (2) use of hormonal contraceptives in women to minimize fluctuations in the sense of smell across the menstrual cycle^92^. Exclusion criteria were: (1) self-reported history of neurological, psychiatric or other disorders that are known to affect olfaction^93^ or sleep^94,95^, with anxious and depressive tendencies being a prime example; (2) suffering from any medical condition at the present time; (3) a history of head trauma with unconsciousness; (4) taking medication with known effects on olfaction or sleep^93^; (5) persistent olfactory complaints^96,97^; (6) mild or more severe depressive tendencies, as evidenced by a score above the minimal range of 0–13 points^98^ on the Beck Depression Inventory II (BDI-II;^99,100^); (7) trait anxiety of potential clinical significance, as suggested by a score greater than 39^101^ on the State-Trait Anxiety Inventory (STAI X-II;^102^); (8) tobacco dependence beyond light smoking, defined as more than 90 cigarettes a month^103^; (9) alcohol dependence beyond moderate drinking, defined as more than one drink per day in women and two drinks per day in men, where a “drink” is 0.33 l of beer, 0.2 l of wine, or 0.02 l of liquor^104^; (10) a history of substance abuse, particularly illicit drug use; and (11) pregnancy or breastfeeding in women. Volunteers were recruited via posts advertising the study on the website and Facebook page of the National Institute of Mental Health (NIMH-CZ) and Faculty of Humanities, Charles University (FHS UK). Compliance with the above-specified criteria was pre-screened with a Qualtrics questionnaire battery (Qualtrics, Provo, UT). Individuals who had indicated their preliminary interest in study participation were e-mailed a link to the anonymous survey, which they completed using a self-generated ID. Eligibility for study participation was evaluated by the researchers and the results were communicated to the respondents within two weeks. Descriptive statistics of these variables are shown in Table 1. Respondents found eligible and confirmed their ongoing interest in study participation were invited to make an appointment with the in-house physician (EM), who confirmed good general health status and referred them as healthy volunteers to the sleep laboratory.

The sample size was calculated a priori based on our previous study with similar design, in which a generally liked and disliked odor was used^90^. The mean difference between the control and exposure condition in dream pleasantness and emotional charge, averaged positive and negative emotions, and core affect ratings was about 1, with an SD of about 1.5. We aimed for a power (1-β) of 0.8, generally recommended by Cohen^105^. At a significance level (α) of 5% and power (1-β) of 80% (two-sided test), the estimated sample size was 20 pairs. However, as the actual valid N depends on the dream recall rate, which was 83% in^90^ but only 75% in the present study, and we encountered technical problems during the v-PSG recordings, fewer reports were available for analysis. Due to time constraints and a tight lab schedule, we could unfortunately not make up for the data loss within the time slot dedicated to this study.

#### Ethics statement

All of the procedures followed were in accordance with the ethical standards of the responsible committee on human experimentation (institutional and national) and the Helsinki Declaration of 1975, as revised in 2008. The study protocol was approved by the Institutional Review Board of the National Institute of Mental Health as part of a larger project on psychophysiological correlates of dreaming under all-night ambient odor exposure (Approval No. 57/16, amended by Approval No. 5/18). Written informed consent was obtained from the participants. Upon study completion (i.e., on their third and final visit), the participants received a financial compensation of 1500 CZK (app. 58 EUR).

### Questionnaires

#### Health- and sleep-related screening inventory

The pre-screening battery consisted of a brief inventory to evaluate the respondents’ family and personal medical history, including neuropsychiatric and neurological diagnoses, past admissions to medical facilities for treatment, medication used in the previous six months, sleep habits, and consumption and abuse of substances known to affect olfaction and/or sleep (e.g., nicotine, caffeine, alcohol, illicit drug use). This data was used to evaluate compliance with exclusion criteria.

#### Beck Depression Inventory-II (BDI-II)

Depressive tendencies, which were part of the exclusion criteria, affect olfactory perception^106,107^ and sleep^108,109^ and may contribute to the so-called first-night effect^110,111^, whereby sleep tends to be distorted during the first nocturnal assessment at the sleep laboratory^112,113^. They are also linked to intense negative reactions to even weak everyday odors^114,115^.

To briefly evaluate depressive tendencies, we used the 21-item BDI-II^100^, published in Czech by Preiss and Vacíř^99^. Within each item, respondents are presented with a list of four graded statements, for example, “I did not have any thoughts of killing myself”; “I have had thoughts of killing myself, but I would not carry them out”; “I would have liked to kill myself”; “I would have killed myself if I had the chance” and asked to select the one that best reflected how they had been feeling in the past two weeks. The statements are always weighted from 0 (not present) to 3 (severe), with higher scores indicating greater severity. Themes assessed by the instrument include sadness, pessimism, past failure, loss of pleasure, guilty feelings, feelings of punishment, self-dislike, self-criticalness, suicidal thoughts or wishes, crying, agitation, loss of interest, indecisiveness, feelings of worthlessness, loss of energy, changes in sleeping patterns, irritability, changes in appetite, difficulties concentrating, tiredness or fatigue, and loss of interest in sex. The individual weights are summed to produce the total score with a theoretical range of 0 to 63 points. The higher the total score, the greater the depressive tendencies. Only respondents scoring 0–13 points were deemed eligible for participation in the present study.

#### State-Trait Anxiety Inventory (STAI X-II)

Anxiety is another factor that may play a role in the first-night effect^116,117^. Furthermore, trait anxiety is likewise linked to various alterations and impairments of chemosensory processing^118–120^ and intense negative responses to common odors^115,121^.

To screen for anxious tendencies, we employed the validated State-Trait Anxiety Inventory (STAI;^122^). Its two forms are widely used in research and clinical practice to assess state (form X–I) and trait (form X–II) anxiety. To rule out an anxious disposition that is of clinical interest and might interfere with experimental procedures, we asked our participants to complete the trait scale, which has nonetheless been found also to encompass depression in addition to anxiety^123^. The trait scale consists of 20 statements, such as “I feel pleasant” or “I wish I could be as happy as others seem to be”, and respondents are asked to rate each of them with regard to how they *generally* feel. Ratings are provided using a four-point scale (almost never/sometimes/often/almost always). Positive items must be reverse-coded before calculating the total trait anxiety score, which may range between 20 and 80 points. Higher scores are indicative of greater tendencies towards anxiety, with scores over 39 suggesting anxiety of clinical significance^101^. The Czech translation of the restandardized version was used^102^.

#### Dream characteristics and emotions inventory

On awakening, the participants were asked to complete a custom-designed inventory to assess several affective properties of the mental sleep experience they may have had during the preceding sleep period. The participants were first asked whether they had been dreaming (yes/no), and if so, how pleasant and emotionally charged their experience was on a 7-point scale anchored with 1 = “extremely unpleasant” and 7 = “extremely pleasant” and 1 = “not at all” and 7 = “to a great extent”, respectively. Further, they rated the presence of selected positive and negative emotions in their dreams on a 7-point scale (1 = “not at all”, 7 = “to a great extent”). The selection of the four most common positive dream emotions (i.e., joy/happiness, love, contentment, and interest/excitement) was based on the work of Fredrickson^124^ and that of the negative ones (i.e., anger, apprehension/fear, sadness, and confusion/shock) on Domhoff^125^. No definition of dreaming or dream emotions was provided, and the participants did not request any clarifications of the terms used. The individual emotion ratings within either category (i.e., positive and negative) were averaged across each participant, with higher mean scores indicating greater magnitude of positive and negative emotions experienced in sleep mentation.

#### Swedish Core Affect Scale: Valence dimension index

To measure the current affective state, we asked the participants to complete the Swedish Core Affect Scale^126^. Russell^127^ defines core affect as “a neurophysiological state, accessible to consciousness as a simple nonreflective feeling: feeling good or bad, feeling lethargic or energized.” It is not synonymous with “emotion”, which begins and ends^128^; rather, it somewhat resembles “mood” because it lasts and varies over time along with the activity of the autonomic nervous system, cognitive processes, and behavior^129^. Although authors differ in their views of how many dimensions of core affect there are^126,130–132^, in subjective perception, these dimensions fuse to produce “a single integral blend”^129^. One of the main dimensions of core affect is Valence, also referred to as positive—negative hedonic tone, pleasure—displeasure or pain, approach—avoidance^133,134^. Positive valence means feeling pleased, glad, and happy rather than displeased, sad, and depressed. The Valence dimension comprises three adjective pairs, each rated on a nine-point scale. For instance, if one is feeling as displeased as they can be, they will place their response as close to the negative end of the scale as possible and receive a score of “1” (and similarly for the other two adjective pairs that constitute the valence dimension, i.e., feeling sad—glad and depressed—happy). The outcome is the dimension index, calculated as Σ (weight x adjective scale rating), where the weight equals 1.

#### Other instruments

The participants were instructed to follow basic sleep hygiene recommendations^135^. Compliance was verified with a sleep diary, described in detail in Martinec Nováková et al.^90^. The participants kept the diary throughout the 14 days (from the check-in on their first visit to the check-out on the morning of their third (and final) visit). Relatedly, at the check-in of each visit, they also provided assessments of sleep inertia typically experienced over the past week, which may indicate chronic sleep deprivation^136^ and is characterized by impaired performance, lowered vigilance, drowsiness, sluggishness, and a desire to go back to sleep^137^. This was done using the 21-item Sleep Inertia Questionnaire (SIQ;^138^); see Martinec Nováková et al.^90^ for a detailed description.

### Olfactory assessment

Normal olfactory function was ascertained with the Czech version of the 40-item self-administered University of Pennsylvania Smell Identification Test (UPSIT)^139^. Being a “scratch and sniff” test, this standardized tool consists of 4 booklets, each containing ten chemically microencapsulated odor patches that release an odor when rubbed with a pencil tip. Four possible verbal labels are provided for each odor, of which one is the target label, and three serve as distractors. For each correct choice, the participant earns one point, and the individual scores are summed to produce the total odor identification score, which may range between 0 and 40. Normative data for the Czech population are not available, but in the US population, scores greater than 31 in men and 30 in women are thought to be indicative of normosmia^140^. The test–retest reliability of UPSIT exceeds 90%^139^, and the completion time is about 20 min.

### Olfactory stimulation

Each participant was presented with a set of 40 scented cartridges. They were placed at random in a box. The participants were instructed to retrieve them at random, one by one, sniff at each one of them, and finally pick whichever reminded them of something pleasant and intensely positive. They were told to take as much time as needed, proceed at their own pace and complete the task anytime within the circa four-hour time frame (from check-in at 6 p.m. to the start of the session at 10 p.m.). The task was introduced as follows: “This box contains 40 fragrant cartridges. Please smell them all, one by one, and then choose one whose smell evokes intense positive memories of specific people, places, events, periods, etc. If you cannot pick just one of them, please select the one that affects you emotionally the strongest. If none of them elicit any positive memory or emotion, please choose one pleasant enough to consider dispersing in your own bedroom.” The stimuli were designer room fragrance cartridges, manufactured by Mr & Mrs Fragrance or Kartell Fragrances and distributed in the Czech Republic by Joy Fragrances, that were compatible with the home aroma diffuser that was used for odor dispersion (Otello, Mr & Mrs Fragrance). To mimic the real-life context of scented product choice^141^, we presented the cartridges in their original packaging, allowing the participants to appraise, along with the scent itself, the visual identity of the product (e.g., amber-hued cartridges housed in a sleek gold-and-black paper box), its name (e.g., “Alhambra”), declared scent character (e.g., “citrus, floral, oriental, woody”), and chemical composition (e.g., “tangerine, lime, jasmine, amber, praline, patchouli, cedarwood”). Multiple odor—participant pairings were allowed, and, as Table 3 suggests, several odors were picked by more than one participant. This was even though the position of the individual cartridges within the box was randomized each time before a participant was invited to engage with the stimuli. Next, the participants were instructed to rate the selected scent for pleasantness, intensity, and familiarity. To do so, they were asked to use a 9-point scale, with higher ratings suggesting greater pleasantness, intensity, and familiarity.

**Table 3:** Perceptual characteristics (pleasantness, intensity, and familiarity) and mean ratings on the three factors of the Semantic Differential (SDiff), i.e., Evaluation, Potency, and Activity, for the 14 odors used in the study. N > 1 suggests more than one participant selected the odor.

Odor	Name	Scent composition	N	Perceptual Characteristics			Semantic Differential Factors			Description of mental associations or imagery
				Pleasantness	Intensity	Familiarity	Evaluation	Potency	Activity	
1	Hawaiian Poppy	Orange, bergamot, mint, poppy, cyclamen, rose, lilac, woods, musk	1	8	3	2	1.3	5.7	3.8	
8	Florence Talcum Powder	Sicilian lemon, bergamot, pink pepper, iris, violet, rose, cotton plant blossom, cedar wood, vanilla, musk	1	9	6	9	2.1	5.0	3.8	Lipstick shared with a friend at high school
19	Asian Vervain	Mandarin, verveine, lemon blossom, lily of the valley, aromatic herbs, juniper, musk	1	7	8	7	3.1	5.3	3.8	
20	Hokkaido Lavender	Lavender, orange, patchouli, citronella, geranium, tonka bean, sage, fern, musk	1	6	7	9	2.8	1.7	3.8	Scent of foaming bath their mother used to prepare for them as young children, bathroom games with brother
31	Citronella & Flowers	Lemongrass, lemon, elemi, rose, ylang-ylang, guaiac wood	1	8	8	9	2.1	1.7	4.8	Working as a tour guide at the Kuks hospital, roaming alone its monumental corridors decorated in frescoes, sunlit and deserted after visiting hours, with a geranium-like floral scent lingering in the air; the smell of its baroque pharmacy and the various herbs, medicines, and ointments of bygone days; a sense of solitude, stillness, and being surrounded by history
32	Parsley & Tomato	Sparkling parsley, tomato leaves, spring nettle, vegetable garden	1	7	7	7	2.6	4.7	4.6	Smell of ex-girlfriend’s place
34	Citronella & Mint	Lemon, litsea cubeba, mint, lemongrass, orange	1	6	8	5	4.2	1.3	6.0	A month-long vacation spent in Australia at the age of 11, scent of the hotel room
37	Undercover	Plum, jasmine, orange blossom, honey, woody notes, sugar	1	8	8	7	2.8	3.3	4.4	
38	Citrus & Herbs	Citrus, sage, mint, lily of the valley, freesia, sandalwood, musk	1	6	6	7	2.3	5.3	3.8	
42	Lavande Naturelle	Lemon, bergamot, galbanum, basil, clove, lavender, sage, artemisia, oakmoss, patchouli, pine	2	8.5	7.5	9	1.9	2.2	2.3	P1: Endless Mediterranean lavender fields, sunny days spent at the sea, sipping lavender lemonade with friends; P2: Herbal scent promoting relaxation, fragrant bath salt used to unwind after a busy day, resting in a scented steam-room, soothing herbal tea, a sense of peace and cleanliness, enjoying “me” time
44	Temptation Ave	Bergamot, anise, jasmin sambac, rose, sandalwood, amber, vanilla, caramel, tonka beans, musk	4	7.8	7.5	5.3	2.0	1.6	3.1	P1: Cotton candy, fair, childhood; P2: Cotton candy, cherry, fair, scented candle gifted by boyfriend, time spent with boyfriend; P3:
46	Portofino	Bergamot, marine accord, tangerine, green lemon, lavender, rosemary, jasmine, cedarwood, patchouli, amber accord	1	9	7	9	1.6	3.3	4.2	Freshly showered boyfriend
47	Alhambra	Tangerine, lime, jasmine, amber, praline, patchouli, cedarwood	3	8.7	5.7	4.7	2.3	3.6	2.5	Some scented product a friend used to wear
48	Noir	Amber, incense, turkish rose, ylang-ylang, jasmine, cardamom, cumin, atlantic white cedar, oak musk	1	6	9	7	4.4	1.3	4.2	Fragrance gifted for Christmas by a high school classmate that was only used on special occasions such as dance classes, prom night, and afterparty. Reminds him of his classmate, then-girlfriend, and lost high-school friends. Evokes a sense of “sweet melancholy” and nostalgia

The ratings of odors 42, 44, and 47 are averaged scores. Higher perceptual ratings indicate greater pleasantness, intensity, or familiarity. Evaluation SDiff scores ranging between 1 and 3 suggest that the stimulus was rated as fresh, good, happy, harmonious, healthy, beautiful, smooth, clean, and safe rather than stale, bad, sad, unharmonious, unhealthy, ugly, rough, dirty, and dangerous. Evaluation scores in the range of 5–7 suggest the opposite. Potency SDiff scores < 4 indicate that the odor was evaluated as strong, powerful, and harsh rather than weak, powerless, and mild, whereas ratings above 4 suggest the contrary. Activity SDiff ratings ranging between smaller than 4 mean that the stimulus was perceived as more ordered, quiet, relaxing, wet, and muddy than chaotic, noisy, stimulating, dry, and clear, while scores over 4 indicate the opposite. P1, P2, etc. denote Participant 1, Participant 2, etc., reports for odors picked for stimulation more than once.

#### Semantic Differential

**Figure 1:**
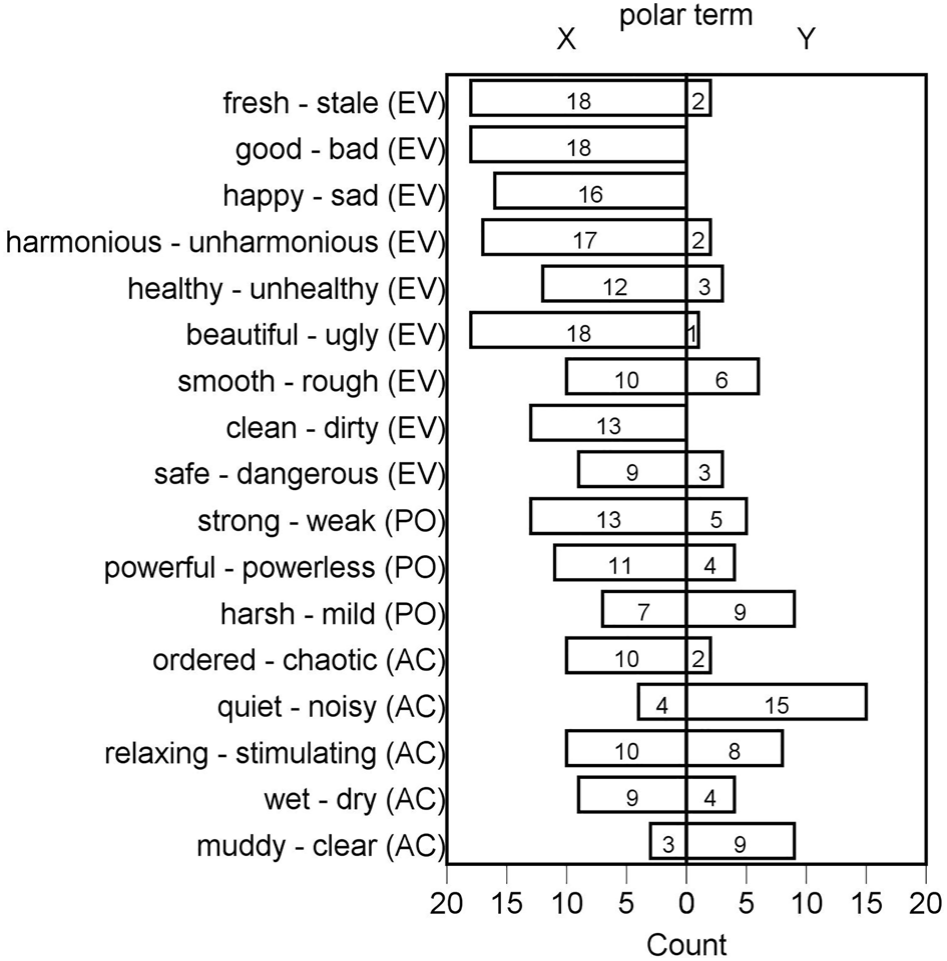
The frequencies of endorsement of the individual polar terms comprising the 17 pairs of adjectives of the olfactory Semantic Differential to evaluate the self-selected, preferred stimuli. Responses “slightly”, “quite”, and “extremely” on the given side of the scale were grouped. Responses “neither X nor Y” and “equally X and Y” are not shown. The left column represents the frequency of the first polar term within the pair, and the right column shows the frequency of the other polar term. For example, eighteen participants felt that the odor of the cartridge they selected was “good” and none thought it was “bad”. AC = Activity, EV = Evaluation, PO = Potency dimension of the olfactory Semantic Differential.

To achieve a more detailed characterization of the stimuli and obtain information about the semantic and affective dimensions that underlie the participants’ mental representations of the odors, we used the Semantic Differential technique (SDiff; ^58^). Odor SDiff ratings (e.g.,^36^) are used to capture the complexity of the sensory and affective experience of smelling odors while reducing reliance on verbal descriptions, which are notoriously difficult to provide for odors^142^. The participants were presented with 17 adjective pairs, which, in the study of Dalton et al.^58^ loaded onto three dimensions: Evaluation (nine pairs; e.g., good–bad, happy–sad, healthy–unhealthy), Potency (three pairs; strong–weak, powerful–powerless, harsh–mild), and Activity (five pairs; e.g., quiet–noisy, relaxing–stimulating, wet–dry). Each adjective pair was rated on a 7-point scale. A label was placed at each of the scale points, so that the point closest to polar term X was labeled “extremely X”, the next one “quite X”, and the following one “slightly X”. The center of the scale was marked “neither X nor Y; equally X and Y”. Moving towards the opposite polar term Y, the labels followed in reverse order. Ratings on each dimension were averaged across the relevant scales, with lower mean scores suggesting ratings on the given dimension were closer to polar term X than Y. The frequencies of endorsement of the paired polar terms are shown in Fig. 1.

#### Room odorization and its appraisal

To mimic all-night exposure to ambient odor at home as closely as possible, we utilized a commercial home aroma diffuser (Otello, Mr & Mrs Fragrance). The participant-selected cartridge was used on exposure (stimulation) nights, while on odorless nights, the diffuser operated with no cartridge. Since the diffuser does not generate heat, steam, vibration, etc., and only produces a faint humming sound (about the intensity of a notebook fan), it could be positioned under the bed next to the headboard. The diffuser can be run in several operation modes. One of them affords continuous odor dispersion for up to ten hours. Hence, on stimulation nights, it would be switched on at 10 p.m. and off at the participant’s departure after 7 a.m. to prevent excessive odor dispersion that would require a prolonged ventilation period to get the bedroom ready for subsequent use. On control nights, the empty diffuser was left to run until the staff serving the next shift came to remove it from the bedroom. The air ventilation system in the sleep laboratory was turned off between 10 p.m. and 7 a.m. on both conditions to prevent noise caused by its operation.

The participants were asked to appraise ambient odor in exposure and control conditions. If they indicated that they had noticed *any* smell in the room, they were asked how pleasant, intense, and familiar it felt on a nine-point scale. In the exposure condition, affirmative responses were considered “hits”; in the control condition, they were regarded as “false alarms”. Negative responses were categorized as “misses” and “correct rejections”, respectively. Nevertheless, the question's phrasing raises the possibility that affirmative responses referring to background odors rather than the stimuli were erroneously classified as “hits”.

### Video-polysomnography and sleep stage identification

To identify a waking point from which interim reports could be elicited across all the participants, we asked the participants to undergo overnight video-polysomnography (v-PSG; M&I, Brainscope, Czech Republic). V-PSG is the systematic collection of physiologic parameters during sleep^143^. We utilized electroencephalography (EEG; F3/A2, F4/A1, C3/A2, C4/A1, O1/A2, and O2/A1 leads), electrooculography (EOG), mental and tibialis anterior electromyography (EMG), electrocardiography (ECG), and pulse oximetry; recorded nasal-oral air flow, oropharyngeal sounds, thoracic and abdominal efforts (via belts), and also obtained synchronized video and audio recordings to determine sleep stages. The sleep stage that was of interest to the present study was the rapid eye movement (REM) stage, which is a physiological state characterized by fast EEG activation with dreaming, cardiovascular changes, muscle atonia, higher arousal threshold, and other features^144^. Dreaming is by no means limited to REM sleep^54^. Still, REM mental processing may qualitatively differ from non-REM^55^, particularly in the greater involvement of REM sleep in processing emotional material^56^. Sleep stage scoring was performed according to international criteria^145^ by JB, who was blind to the participants’ randomization status.

### Procedure

Eligibility was pre-screened with the health- and sleep-related screening inventory, BDI-II, and STAI X-II. Those who passed the pre-screening were notified within two weeks and invited to schedule an appointment with the in-house physician. The physical examination usually took place several hours before the first night at the sleep laboratory. The physician verified the participants' good health status and referred them to the sleep laboratory.

Meeting the critical but often-overlooked criterion of aromachological research^59^, namely, that appropriate controls be employed, we required our participants to visit the sleep laboratory repeatedly in weekly intervals on the same day of the week to receive exposure or not. The order of exposure vs. control condition (i.e., on which night the stimulation occurred) was randomized across the participants. Moreover, in sleep studies, it is necessary to also account for the fact that we often experience disrupted sleep in a novel environment^146^. This is a common sleep disturbance, particularly during the sleep-onset period^147^, that occurs regardless of the participant’s state anxiety level^148^. Known as the “first-night effect”^112^, it is usually addressed by incorporating an extra night in the design that is not analyzed and whose primary purpose is to adapt the participants to the sleep laboratory environment and familiarize them with the procedures^149^. Therefore, the first night served as an adaptation visit, and the data from that session were not subject to formal analysis.

On each visit, the participants checked in at the medical sleep clinic/laboratory of the National Institute of Mental Health at around 6 p.m. They were required to stay on the premises until the session commenced at 10 p.m. During that time, they spent about 1 hour in the hall completing check-in forms and questionnaires, about 2 hours in the EEG fitting room, and 1 hour relaxing in their bedroom. During this time, they were under constant surveillance. Upon arriving at the sleep laboratory on the first night, the participants were shown around and familiarized with the procedures. Next, they self-administered the UPSIT and were handed the box with scented cartridges for appraisal, stimulus selection, and rating. They were encouraged to go through the box at their own pace, take time, and take as many breaks as needed. This procedure was specific to the first night only. After that, a sequence of procedures was performed on each visit, which involved fitting the v-PSG equipment and completing SIQ for the past week. No pre-sleep affect assessments were made because state affect, i.e., “what one is feeling at any given moment in time”^150^, and its valence in particular (i.e., negative vs. positive) tends to fluctuate on a momentary and situational basis, and in some people more than in others^151^. As a result, some people’s ratings of current affective state would have been preponderantly, if temporarily, affected by the procedures they had been subjected to at the sleep laboratory, making it chalenging to select a good point for baseline measurement. Instead, the emotional dimension of SIQ was used to briefly evaluate and control for the participants’ prevailing emotional state specifically with regard to sleep and waking over the past week.

The diffuser was placed under the participants' beds shortly before they retired. V-PSG recordings always started at 10 p.m., when participants went to bed, and the lights went off. For the next eight hours, participants were asked to keep their cell phones, notebooks, and lights in the room and the adjacent bathroom switched off. To request assistance from the sleep laboratory staff (e.g., to get unplugged before using the bathroom), the participants were instructed to sit up on the bed and use the intercom.

REM was the sleep stage we pre-specified for waking to collect interim reports of the affective state. REM is involved in emotional memory processing^152^ and is thus conducive to collecting reports of sleep mentation^46,47,51,87,90^, which we then used to control for the effect of dream valence and emotions on post-sleep affect. To this end, after 2:30 a.m., in the monitoring room, the researchers started to visually inspect the v-PSG recording in real time to detect REM. The criteria for REM detection included a desynchronized EEG without spindles or K-complexes accompanied by decreased tonic chin/sub-mental EMG amplitude and rapid eye movements. Once the REM stage hit the five-minute mark, the researchers approached the sleeping participant to wake them up with a pre-arranged signal (e.g., tapping them on the shoulder or calling their name). The participant was temporarily unplugged from the v-PSG equipment and sat at the table with a small lamp to complete the first batch of post-sleep measures. Their completion took about five to ten minutes. The participants first indicated  the presence or absence of any sleep mentation and its emotional charge, then assessed their current affective state, and concluded by appraising the ambient atmosphere. All the assessments were made using paper forms (rather than by interviewing participants via the intercom), which, considering the number of administered measures, has previously proven to be practical^90^. After that, the participant returned to bed, and the v-PSG recording was resumed. It continued until the spontaneous waking in the morning or, if the participant had requested to be woken up, until the pre-arranged waking time. As soon as they got up, the participants again provided evaluations of sleep mentation experienced during the sleep episode that followed the first waking, assessments of post-sleep affect, and appraisal of ambient odor.

A day or two before each scheduled appointment, the participants were reminded of their upcoming visit and asked if they were feeling well. If they had fallen ill, the visit was rescheduled for a later time. On the morning of their third and final visit, the participants were reimbursed with CZK 1,500 in cash. Data collection occurred between September 2018 and March 2019.

### Statistical analysis

The exploratory analyses and plots were produced with IBM SPSS 24.0. The normality of the raw data was checked, firstly, by visually examining the individual histograms of all relevant variables, secondly, by producing skewness and kurtosis values and their respective standard errors, from which z-scores were computed and compared to the value of 1.96, as suggested by Field^153^ and, thirdly, with multiple Shapiro–Wilk’s W tests. Since the data was mostly non-normal and transformations had little effect, non-parametric tests were preferred. To compare the odorless control and odor exposure condition, Wilcoxon signed-rank tests were run. Differences between randomization groups and genders were analyzed using Mann–Whitney U tests. Effect sizes represented by Pearson’s *r* for Mann–Whitney U and Wilcoxon signed rank tests were computed after Rosenthal^154^ as follows: $r=\frac{z}{\sqrt{N}}$.

### Supplementary Information

Supplementary Information.

## Data Availability

The datasets generated and analysed during this study are available from the corresponding author on reasonable request.
