# Supplementary material for: Nocturnal exposure to a preferred ambient scent does not affect dream emotionality or post-sleep core affect valence in young adults
Source: Sci Rep. 2024 May 6;14:10369. doi: 10.1038/s41598-024-60226-z (PMC11074142; doi:10.1038/s41598-024-60226-z)
Supplement: Supplementary file 1 — Supplementary Information. [file 41598_2024_60226_MOESM1_ESM.pdf]

Nocturnal exposure to a preferred ambient scent does not affect dream emotionality or post-sleep core affect valence in young adults

Lenka Martinec Nováková[1,*], Eva Miletínová[2,3], Monika Kliková[2,3], Jitka Bušková[2,3]

[1] *Department of Chemical Education and Humanities, University of Chemistry and Technology, Prague, Technická 5, 166 28 Prague 6 – Dejvice, Czech Republic*

[2] *National Institute of Mental Health, Topolová 748, 250 67 Klecany, Czech Republic*

[3] *3rd Faculty of Medicine, Charles University, Ruská 87, 100 00 Prague 10, Czech Republic*

* Corresponding Author; Email: martinel@vscht.cz

Supplementary Results

1. Dream Recall

Dream recall was a prerequisite for evaluations of sleep mentation characteristics (but not for core affect ratings). On the second visit to the sleep laboratory, the dream recall rate was 84% (N = 16), and on the third (and final), it was 82% (N = 14). The dream recall rate was the same on the control and exposure condition (75%, N = 15). There were no gender differences in dream recall, whether within visits or conditions.

2. Odors, Odorization, and Their Appraisal

2.1. Stimulus Selection

Most participants tended to pick scents with dominant citrus, lavender, and other notes believed to have anxiolytic, sedative, and relaxation-promoting properties (*1*). In line with this, as regards the ratings of the 17 adjective pairs of the olfactory Semantic Differential (*2*), only two participants were unable to decide whether the odor they selected felt rather "relaxing" or "stimulating" (which was one of the pairs that constituted the Activity factor). None of them found that it was either. Ten participants chose an odor that felt to some extent "relaxing", while another eight selected one they perceived as "stimulating".

As expected, on average, the participants evaluated the odors they selected as pleasant (7.70 ± 1.13, range 6 – 9), intense (6.95 ± 1.57, range 3 – 9), and familiar (6.55 ± 2.67, range 1 – 9). Lower familiarity ratings (< 4) were given to odors 1 ("Hawaiian Poppy"), 44 ("Temptation Ave"), and 47 ("Alhambra") and were generated by three of the eight people who picked a scent because it conjured up pleasant imagery but was not associated with any emotionally charged memories.

2.2. Correlates of Ambient Odor Appraisal

In “hits”, pre-exposure scent pleasantness, as initially assessed on study entry before the adaptation night, practically significantly correlated with ambient odor pleasantness in the exposure condition ($\rho = 0.37$, $p = 0.24$, $N = 12$). In the control condition, participants with better odor identification abilities were practically and/or statistically significantly less likely to report the presence of an odor even though they had not been stimulated ($\rho = -0.49$, $p = 0.053$, $N = 16$).

3. Associations of Perceived Odor Presence with Dream and Core Affect Reports

Perceived ambient odor presence (yes/no) was not associated with dream recall on either occasion. However, explorations revealed elevated core affect ratings in reports of perceiving odor in the control condition. Specifically, the three participants who felt they perceived an odor even though they had not been stimulated also tended to provide higher core affect valence ratings than volunteers who correctly rejected odor presence (6.67 ± 1.20, range 5.67 – 8.00 vs. 5.33 ± 0.97, range 4.00 – 7.67; $\rho = 0.49$, $p = 0.054$).

References

1. A. J. Farrar, F. C. Farrar, Clinical Aromatherapy. *Nurs Clin North Am* **55**, 489-504 (2020).
2. P. Dalton, C. Maute, A. Oshida, S. Hikichi, Y. Izumi, The use of semantic differential scaling to define the multidimensional representation of odors. *J Sens Stud* **23**, 485-497 (2008).
